# The Influence of the Scout Movement as a Free Time Option on Improving Academic Performance, Self-Esteem and Social Skills in Adolescents

**DOI:** 10.3390/ijerph17145215

**Published:** 2020-07-19

**Authors:** Jorge Asensio-Ramon, Joaquín F. Álvarez-Hernández, José M. Aguilar-Parra, Rubén Trigueros, Ana Manzano-León, Juan M. Fernandez-Campoy, Carolina Fernández-Jiménez

**Affiliations:** 1 Hum-878 Research Team, Health Research Centre, Department of Psychology, University of Almeria, 04120 Almeria, Spain; jorge@creailusion.com (J.A.-R.); jalvarez@ual.es (J.F.Á.-H.); aml570@ual.es (A.M.-L.); 2 Department of Language and Education, University of Antonio de Nebrija, 28015 Madrid, Spain; 3 Department of Education, University of Almeria, 04120 Almeria, Spain; jfc105@ual.es; 4 Department of Psychology, University of Granada, 18010 Granada, Spain; carolina@ugr.es

**Keywords:** scouting, academic performance, scouts, self-concept, social skills

## Abstract

The word scouting refers to the Scout movement, born more than a hundred years ago, which educates millions of young people between the ages of six and twenty-one in their leisure time. We aimed to study the effects of scouting on the academic results, social skills, and self-esteem of high school youths compared to a non-scout sample. The selected sample consisted of 430 secondary students aged between thirteen and seventeen. Self-esteem and social skills were measured, and the average mark of the total sample was analysed. After the study, it was shown that belonging to the scout movement significantly influences the improvement of academic results in formal education and conflict resolution; however, there are no statistically significant differences in self-esteem and other social skills.

## 1. Introduction

### 1.1. The Scout Movement

Continuous changes in society generate the need to manage non-formal areas of learning as an extension, enrichment and alternative to traditional forms of teaching, understanding that learning and human development not only occur in formal contexts, but extend to an entire sociocultural network of non-formal and informal learning. In this manner, Smitter [1] formulates that education should be promoted and understood as a comprehensive and permanent process, which is fulfilled in each student throughout their life, thus granting a series of personal and social competences and skills, along with values such as independence, discipline and creativity [2].

Of the options available for children and adolescents to carry out extracurricular activities or leisure activities, scouting is included. Scouting is a youth movement based on the learning of values such as solidarity, mutual help and respect. All of the Scouts in the world belong to WOSM (World Organization of the Scout Movement). There are more than 50 million members around the World. The Boy Scouts of America have about three million members. The Global Scout movement comprises national Scout organizations that have been formally recognized by the World Organization of the Scout Movement. Brostrom [3] states that students who have had exposure to scouting achieve greater leadership skills and are able to take on greater responsibilities. Similarly, Du Merac [4] refers directly to the positive effects on students who have taken part in the Scout movement, since they participate in activities where participants socialize with their peer group, their family and their environment, leading to the development of social skills thanks to the interaction and positive management of conflicts in a non-formal educational environment [5]. Finally, Matsuoka [6] describes the Scout movement as an education in nature.

The purpose of the Scout movement is to contribute in the physical, mental and spiritual development of children and adolescents, with activities taking place in nature. Its mission is to “contribute to the education and development of people, mainly during childhood, adolescence and youth, through a system of values based on law and The Scout Promise, to help build a better world” [7]. In the same way ASDE (Scouts of Spain) [8] defines their area of development for student as being spiritual, intellectual, social, physical and affective.

The Scout movement is immersed in non-formal education, which is characterized by providing innovative, diverse and context-specific learning strategies for children, youths and adults around the world [9]. Regarding the methodology used in the Scout movement, Vallory [10] lists the following basic elements: education in values such as respect, responsibility, loyalty, an attitude of service and respect for the environment, through the transmission of the Scout spirit; and learning by doing through project work in each of the sections that make up the movement, depending on age. The project is an activity that defines Scout methodology, a time when the students devise, choose, plan, perform and evaluate educational actions guided by their monitor; development programs focus on the centers of interest of the participants, with a direct contact with nature, with the aim of developing intellectual, social, physical, affective and spiritual areas.

Scouting is therefore a movement based on free time and leisure shared by students and educators. According to the classification carried out by Quintana [11], it would be placed in the category of utilitarian time, free of primary needs and obligations. Oropesa [12] in his analysis of leisure time and its influence on the evolutionary development of the adolescent, presents free time as the ideal context to satisfy the needs of autonomy, competence and relationships with other people which are necessary for the complete development of the adolescent as a person. Included in the full personal development of the adolescent in their free time is the feeling of personal effectiveness, which Bandura [13] defines as coping with tasks in a spirited way, without stress or disappointment.

### 1.2. Academic Performance in Spain

Academic performance is defined by Rodríguez [14] as the results that a student obtains in formal education after completing the curriculum of the subjects that make up formal education. Academic performance is defined as the level of knowledge that students demonstrate in an area or subject compared to a criterion or standard, and this knowledge is measured by the school average [15]. The report “Education Overview 2018. Report for the OECD” of the Ministry of Education [16] declares that 34% of young Spanish people do not reach upper secondary education. Furthermore, the latest PISA (Programme for International Student Assessment) report [17] details that Spain’s school failure among adolescents is high when compared to the European Union average. It is therefore important that both formal and non-formal education reacts with valid proposals for improvement, adapted to the characteristics and needs of the students.

Scouting is a non-formal educational strategy that can positively influence the academic performance of children and adolescents. Recent studies such as that of De Mora [18] establish that scouting, as an after-school activity, is a good predictor of school performance. Campos [19] concludes that non-formal educational activities can enhance the acquisition of key skills in education and their results show that participants in the Scout movement from the age of Cubs (children aged 8 to 10 years) acquired the skills of adaptation, communication, creativity, emotional intelligence, entrepreneurial spirit, leadership, problem solving and decision-making. These abilities are directly related to academic performance, as in addition to overall cognitive skills students must show adequate growth in aspects such as emotional control [20]. The Scout movement offers a non-sedentary lifestyle, committed to personal and social skills, which makes it easier for its participants to obtain better academic performance [21].

### 1.3. Self-Esteem and Social Skills in Scouting

Self-esteem consists of an individual’s assessment of himself, an image forged from an early childhood age. However, this concept is dynamic and is not an unchangeable construct [22]. DuBois et al. [23] affirm that self-esteem is the cornerstone on which the teenager strengthens his personality. Moreover, these authors add that, when self-esteem is defined and improved, there is an improvement in the psychological adjustment of the adolescent and/or the adult. Fulquez [24] states that relationships with others which are evidenced by self-esteem, intervene decisively and essentially in the optimal social, personal and professional functioning of the person [25]. Scouting can improve self-esteem and self-concept, as noted by the results of the recent study by Lynch et al. [26]. Youth development programs, such as scouting, develop positive attributes in adolescents and their positive self-perceptions increase significantly in terms of joy, kindness, reliability and future expectations.

Regarding social skills, Kelly [27] defined these as a set of learned behaviors that individuals employ in the interpersonal situations they face in order to gain or maintain a strengthened relationship with their environment. Caballo [28] establishes social skills as behaviors that allow the individual to develop in an individual or intrapersonal context expressing feelings, attitudes, desires, opinions or rights in line with the situation. According to Martinez-Otero [29], the transition from childhood to adolescence implies the acquisition of more complex social skills for the individual. These social skills can be developed in both formal and non-formal education. Within non-formal education, scouting can be seen as a socio-educational strategy to improve social skills, as it provides participation in youth development programs and a unique context in which to promote positive conflict resolution and aid the relational development of youth [26].

In line with this idea and our study, Bartholomeu et al. [30] are assured that a greater repertoire of social skills improves school performance. Furthermore, Syamsulrizal [31] mentions that the Scout movement improves social skills by ensuring that there are five aspects of student behavior that change for the better through the enjoyment of this free time option: personality, discipline, knowledge, skills and confidence. Similarly, Bakhri and Fibrianto [32] affirm the influence of the Scout movement on social skills, outlining a clear positive relationship between scouting and the social skills of the student.

In this sense, based on the empirical evidence described, the need to study Scouting as a non-formal education strategy for the personal, social and academic development of young people is pointed out. This study aims to give visibility to this strategy and to provide new knowledge about the relationship between the different variables studied within scouting.

The objective of this study has been to determine the influence that the Scout movement has on the students who have enjoyed it, based on non-formal free time education.

## 2. Methods

### 2.1. Participants

The selection of the sample was made for convenience from the scouts of the section aged between 14 and 16 years old (school grades between the third age of the ESO (Educación Secundaria Obligatoria) and the first of the Baccalaureate) from the ASDE Scouts of Andalusia (the main association of scout groups of the Autonomous Community of Andalusia). With these prerequisites, and taking into account that ASDE Scouts of Andalusia, according to their census, have 991 active students in the population range chosen to be studied (between 14 and 16 years old), and that we want the margin of error when estimating the data to be less than 6% and a level of confidence of 95%, we conclude that we need a study that covers at least 215 individuals. A sample composed of 215 Scout students from the Scout Group Unity section of Andalusia was defined, with the requirement that they are taking more than one scouting course. Similarly, a control group of 215 students of similar characteristics to the “scout” sample belonging to several secondary schools in the province of Almería was selected, with the premise that they had never belonged to the Scout movement. Therefore, our final sample is comprised of 430 students studying between the third year of ESO (compulsory secondary education) to the first year of Baccalaureate. Of that total, 215 belong to the Scout sample, as they are active in a group in Andalusia. Of these, 68.8% went to public schools and 31.2% to private schools. In the scout sample were 49.8% boys and 50.2% girls with an average age of 14.82 years (SD = 0.94). The remaining 215 students in the sample do not belong to any scouting group, nor do they have experience of scouting. Of these, 75.8% went to public schools and 24.2% to private schools. In the non-scout sample, were 50.7% of boys and 49.3% of girls with an average age of 14.95 years (SD = 0.90).

### 2.2. Measurements

The Quantitative data was collected through the following questionnaires:

Rosenberg Self-Esteem Scale [33] in its Spanish version Rosenberg’s Self-Esteem Scale. This is one of the most widely used tools for the assessment of global self-esteem. The scale includes ten questions with a 4-point Likert-type answer format, from total disagreement to total agreement, the contents of which focus on feelings of respect and self-acceptance. Half of the items are positively stated and the other half negatively. The results classify the subjects of the sample, based on their scores obtained, with a low self-esteem from 0 to 25, normal from 26 to 29 and high from 30 to 40. The reliability of the scale in the Spanish sample was determined by Vázquez et al. [34], with an internal consistency of α = 87.

Social skills scale, elaborated by Oliva et al. [35]. The overall reliability of this test is Cronbach 0.69, and in addition, for its various factors: Communicative Skills 0.72, Assertive 0.77 and conflict resolution 0.75. This questionnaire includes 12 questions with a 7-point Likert-like answer format, from totally false to totally true.

Finally, in order to check academic performance, differences in the samples were analyzed, comparing average performance in all subjects by using the results from the evaluations at the end of their previous course of study.

### 2.3. Procedure

A comparative study was designed for the two samples of teenage students. The methodology corresponds to an ex post facto research design with a cross-sectional design, since the situation already existed and there was no opportunity to vary the independent variables.

First, the Scout sample was accessed through the ASDE Scouts groups of Andalusia by province and group so that they could learn about the census. In this way the characteristics of the Scout sample were identified in terms of: age, type of center in which they study, and socio-economic situation. After analyzing the 215 Scout individuals, it was decided to select the non-scout sample from two institutes in the province of Almeria with similar characteristics, a public and a private center. In order to make the samples comparable, a secondary school was selected from the capital and another from a village near the province.

Using the procedure described, we achieved two groups of students with very similar characteristics. These common characteristics between the two samples were: 70% with relatives (father or mother) having a skilled job; 2–3% of families with both parents without education; 70–76% public IES students.

Initially, as the students were minors, written authorization was sought from the participants’ relatives. Scout monitors were subsequently informed that the questionnaires would be administered at the beginning of the meeting, then the teens were informed that they would be participating in research related to the benefits associated with their participation in scouting. Responses to questionnaires were anonymous. This study was conducted in accordance with the recommendations of the American Psychological Association. The experiment was conducted in accordance with the Helsinki Declaration. Ethical approval was obtained from the Research Ethics Committee of the University of Almería, Spain (Ref. UALBIO 2019/014).

### 2.4. Data Analysis

Descriptive statistical analyses, bivariate correlations and reliability analyses were performed using the SPSS 25 statistical program. In addition, a structural equations model (SEM) was made with the statistical program AMOS 20.

**Figure 1:**
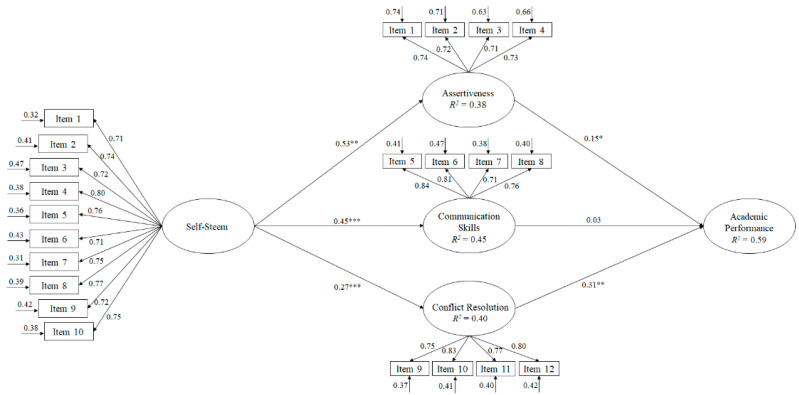
Hypothesized model, where all variables are related to one another. All parameters are standardized and are statistically significant. Note: *** *p* < 0.001; ** *p* < 0.01; * *p* < 0.05

The maximum likelihood estimation method was used to develop the hypothesized model (Figure 1) along with the bootstrapping procedure. To judge the tested model, various adjustment rates were considered: χ2/df, CFI (Comparative Fit Index), IFI (Incremental Fit Index), RMSEA (Root Mean Square Error of Approximation) plus its 90% confidence interval (CI) and SRMR (Standardized Root Mean Square Residual). Values of 0.95 and VALUES of χ2/df less than 3, values for incremental indexes (IFCs, IFIs) close to or greater than 0.95 and RMSEA and SRMR values less than or very close to 0.06 and 0.08 were considered, respectively, as indicative of an appropriate fit of the model to the data [36]. However, Marsh et al. [37] claim that these cut-off values should be interpreted with caution since they can turn out to be too restrictive and difficult to achieve when testing complex models.

For objectives related to the comparison between scout and non-scout groups, student t-tests were used for independent samples. In addition, they were completed along with the calculation of the size of the effect through Cohen’s d.

Finally, a Manova multivariate analysis was performed to determine if there were differences in the scout sample based on time spent in Scouts, gender or age, in relation to the variables studied.

## 3. Results

### 3.1. Preliminary Analysis

Table 1 shows the mean and standard deviation, the bivariate correlations and the analysis of reliability through Cronbach’s α of the variables: self-esteem, assertiveness, resolution of conflicts and communication skills. Regarding the correlation analyzes, these reflected a positive correlation between each of the study variables, except for the correlation between communication skills and academic performance (Table 1).

### 3.2. Analysis of Structural Equations Model

In response to the first objective of the study, to determine the relationships between all the study variables through an SEM, we can point out that the hypothesized predictive relationship model (Figure 1) showed that the adjustment indices were adequate: *χ2* (225, N = 430) = 768.72, *χ2/df* = 3.42, *p* < 0.001, IFI = 0.95, CFI = 0.95, RMSEA = 0.064. (IC 90% = 0.057–0.068), SRMR = 0.039.

These results were adjusted to the established parameters, so the proposed model was accepted as adequate. Similarly, the contribution of each of the factors to the prediction of other variables was examined using standardized regression weights.

Afterwards, in relation to analyzing the influence of scouting on the levels of self-esteem in adolescents, we could point out that no statistically significant differences were found in terms of self-esteem, either in direct or centile scores (Table 2). However, young people belonging to the scout movement seem to have a higher average.

To determine whether scouting influences the academic performance of the students who practice it compared to those who do not, we can see how there are statistically significant differences in academic performance, valued through the average grades of the students, in favor of those that practice scouting, with quite a wide difference, as shown in Table 3. The size of the effect on the same line through the d of Cohen corroborated that the differences between the groups is high.

As can be seen in Table 4, in which the fourth objective is answered (assessing whether scouting improves the social skills of Scout adolescents compared to non-Scout adolescents), we can see how there are statistically significant differences only in the conflict resolution factor with greater ability in favor of scouts. In all cases, the differences are non-existent or small between the groups, as confirmed by the d of Cohen.

Finally, a multivariate analysis was developed to assess whether there were differences based on age, gender, the number of years they had been in the Scouts and level of education and professional qualifications of the families. The MANOVA inferential analysis concluded that there were no statistically significant differences due to age [*p* = 0.752, F (8000) ≥ 1, Lambda de Wilks = 0.987; η^2^ = 0.006]. Neither were there significant differences according to sex [*p* = 0.315, F (2000) = 1.157, Wilks’ Lambda = 0.994; η^2^ = 0.006], nor were they significant in terms of years in the Scouts [*p* = 0.110, F (6000) = 1.733, Wilks’ Lambda = 0.974; η^2^ = 0. 013], nor by reason of family qualification [*p* = 0.731, F (4000) ≥ 1, Wilks’ Lambda = 0.995; η^2^ = 0.002], nor by family educational level [*p* = 0.053, F (6000) = 2.083, Wilks’ Lambda = 0.971; η^2^ = 0.015], nor were there differences in the interaction of the different variables (*p* > 0.05).

## 4. Discussion

This study has examined how scouting can influence the academic performance, self-esteem and social skills of the adolescent population. The results confirm that teens who practice scouting compared to the group of adolescents who do not practice it, have better academic performance and conflict resolution. These findings are consistent with previous studies that have shown that non-formal education, and specifically the scout movement, positively influence personal and social skills of children and adolescents [38]. Lamoneda et al. [39] emphasize that adventure education and scouting promote cooperation between students and the development of learning resources for life in adult society. On the other hand, the positive relationship between the scout movement and academic performance is also recognized through the development of discipline, confidence and greater motivation towards learning [40].

Socio-educational activities in leisure and free-time, such as the Scout movement, aim to promote activities that directly involve youths in decision-making, planning and concrete objectives, which is why young people perceive an increase in their sense of empowerment and self-efficacy [41], and consequently to improve their self-esteem, a fact that has not been evidenced in our study. However, it is important to note that the assessment of self-esteem and self-concept in adolescents may be seen to vary depending on change in mood and current relationship with peer group, especially given that adolescence is a stage marked by major changes and a great vulnerability to contextual developmental changes [42].

Regarding the limitations of our study, we can point out that the sample size was relatively small, so in future research it is recommended that a larger sample is used. In addition, it would be ideal to track, or longitudinally evolve in later studies, young people who join the Scouts and compare them with a control group.

## 5. Conclusions

The study considers that the results obtained are relevant because they provide current data on the scout movement in Spain. In this sense, the data reported in this study is the first step in an area of research that seeks to investigate the feasibility and effectiveness of the implementation of scouting as a non-formal educational strategy to improve personal and social skills, and in this way reduce psychological problems in adolescence that can lead to having low self-esteem, which have a highly negative impact on academic performance and psychosocial development [43]. This suggests the relevance of generating future research that explores the relationship between the scouting movement and interpersonal, psychological, and academic variables.

## Figures and Tables

**Table 1:** Descriptive statistics and correlations between all variables.

Factors	*M*	*SD*	α	1	2	3	4	5
1. Self esteem	3.15	0.54	0.86	-	0.28 ***	0.25 ***	0.24 ***	0.33 ***
2. Assertiveness	4.65	1.09	0.81		-	0.52 **	0.36 **	0.14 **
3. Communication Skills	4.31	1.19	0.83			-	0.15 ***	−0.12
4. Conflict Resolution	5.18	1.11	0.79				-	0.17 ***
5. Academic Performance	6.65	1.98	-					-

Note: *** *p* < 0.001; ** *p* < 0.01. M = Mean; SD = standard deviation; α = Alpha de Cronbach.

**Table 2:** *t*-test to contrast self-esteem levels between scout and non-scout teens.

**Self-Esteem TS**	***M***	***SD***	***t***	***p***	***d***
Scout Group	31.93	4.740	1.580	0.115	0.151
Control Group	31.12	5.884			
**Self-esteem CT**	***M***	***SD***	***t***	***p***	***d***
Scout Group	52.53	27.547	1.202	0.230	0.115
Control Group	49.12	31.302			

TS: Total Sum; CT: Centile Total.

**Table 3:** *t*-test to compare academic performance between Scout and non-Scout adolescents.

**AG**	***M***	***SD***	***t***	***p***	***d***
Scout Group	7.568	1.2878	10.914	0.000	1.050
Control Group	5.724	2.1166			
**AG Rank**	***M***	***SD***	***t***	***p***	***d***
Scout Group	3.27	0.546	8.275	0.000	0.808
Control Group	2.70	0.834			

AG: Average Grade.

**Table 4:** *t*-test to contrast social skills between Scout and non-Scout adolescents.

**TCS**	***M***	***SD***	***t***	***p***	***d***
Scout Group	20.57	7.144	0.007	0.995	0.001
Control Group	20.56	7.138			
**CCS**	***M***	***SD***	***t***	***p***	***d***
Scout Group	51.88	29.629	0.016	0.987	0.001
Control Group	51.84	29.789			
**TA**	***M***	***SD***	***t***	***p***	***d***
Scout Group	16.58	2.958	0.969	0.333	0.092
Control Group	16.30	3.114			
**CA**	***M***	***SD***	***t***	***p***	***d***
Scout Group	49.19	26.105	0.932	0.352	0.090
Control Group	46.79	27.201			
**TCR**	***M***	***SD***	***t***	***p***	***d***
Scout Group	20.20	4.815	2.789	0.006	0.289
Control Group	18.98	4.263			
**CCR**	***M***	***SD***	***t***	***p***	***d***
Scout Group	60.81	28.624	2.789	0.006	0.268
Control Group	53.37	26.676			
**TS**	***M***	***SD***	***t***	***p***	***d***
Scout Group	57.35	10.308	1.547	0.123	0.149
Control Group	55.84	9.948			
**TC**	***M***	***SD***	***t***	***p***	***d***
Scout Group	54.23	29.466	1.117	0.265	0.107
Control Group	51.12	28.365			

TCS: Total sum of communication skills; CCS: Centile communication skills; TA: Total assertiveness sum; CA: Centile assertiveness; TCR: Total conflict resolution sum; CCR: Centile conflict resolution; TS: Total sum; TC: Total centile.
